# Supplementary material for: Human Placental Mesenchymal Stem Cells and Derived Extracellular Vesicles Ameliorate Lung Injury in Acute Respiratory Distress Syndrome Murine Model
Source: Cells. 2023 Nov 29;12(23):2729. doi: 10.3390/cells12232729 (PMC10706384; doi:10.3390/cells12232729)
Supplement: Supplementary file 1 [file cells-12-02729-s001.zip › Supp. figures.pdf]

Supplementary Figures

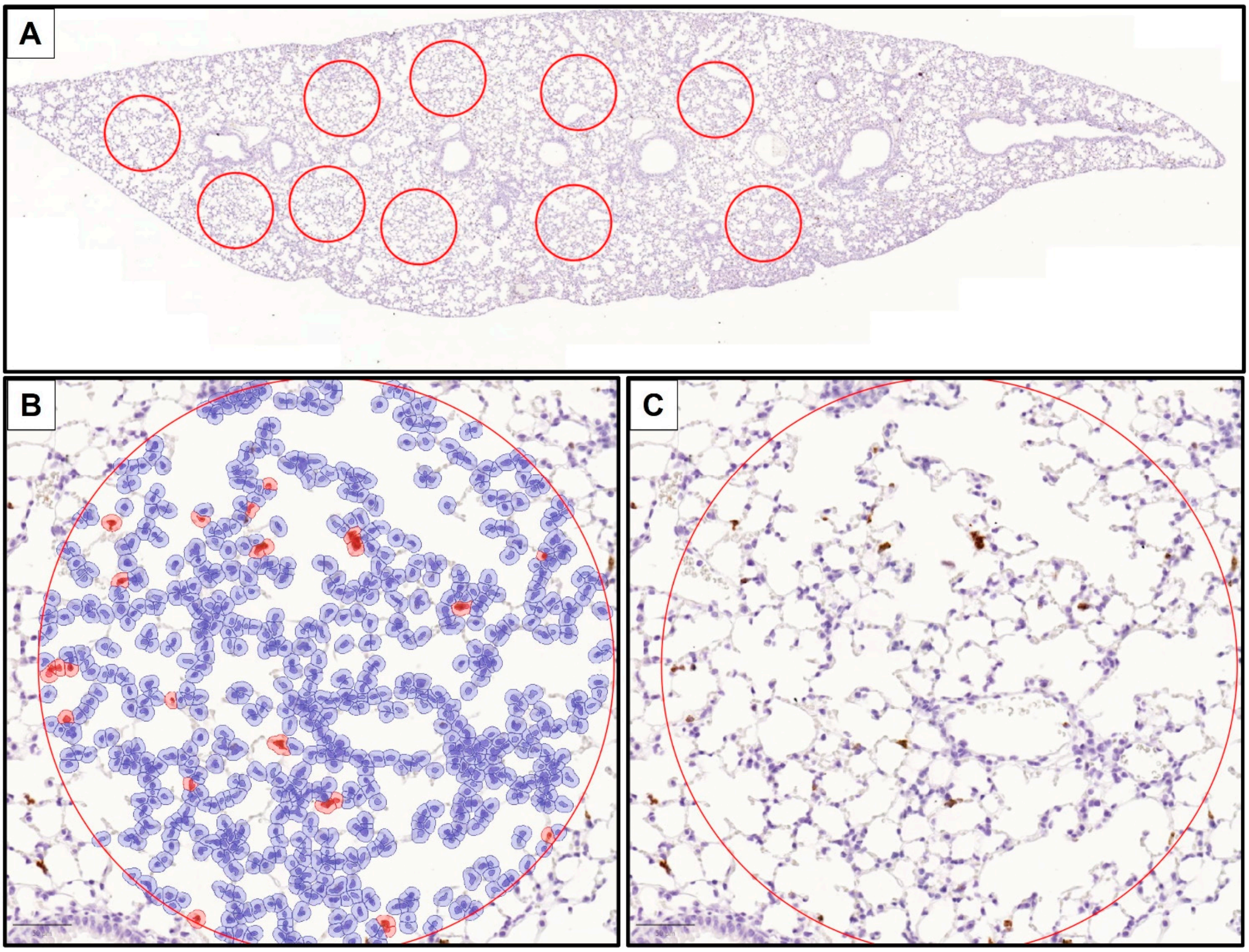

Supplementary Figure S1. Slide annotation and positive cell detection example in a healthy mouse lung section. **(A)** Annotation placement example. Ten 500 µm diameter circle annotations are shown distributed across the lung section, avoiding larger airways, any scanning artifacts, and larger blood vessels. **(B)** Myeloperoxidase (MPO) positive cell quantification with overlay on. Blue cells represent negative cells, and red cells - MPO positive cells. The nuclei are shown in a darker color. **(C)** Overlay off.

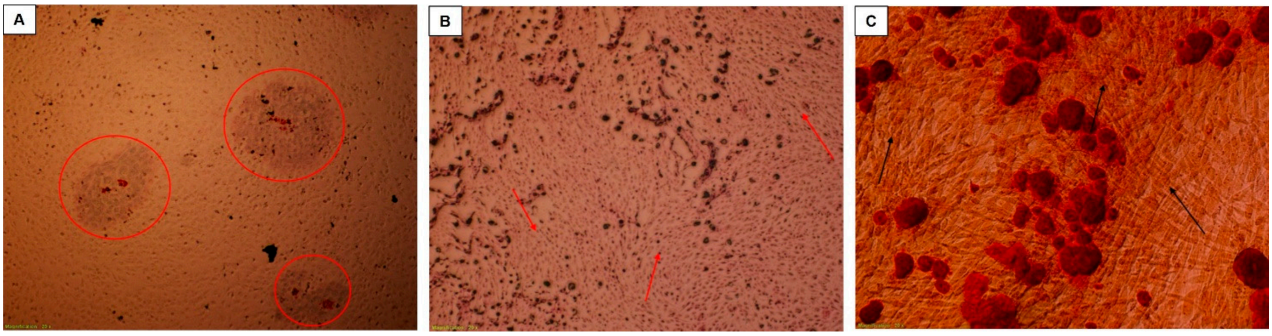

Supplementary Figure S2. Differentiation assay of placental mesenchymal stem cells (20X magnification). **(A)** Evaluation of adipogenesis. Areas of triglyceride producing cells are encircled (Oil-red O stain). **(B)** Evaluation of chondrogenesis. Chondrocyte-like cells producing mucopolysaccharides and glycosaminoglycans are stained violet (Alcian Blue stain). **(C)** Evaluation of osteogenesis. Calcium producing cells are stained red (Alizarine Red stain).

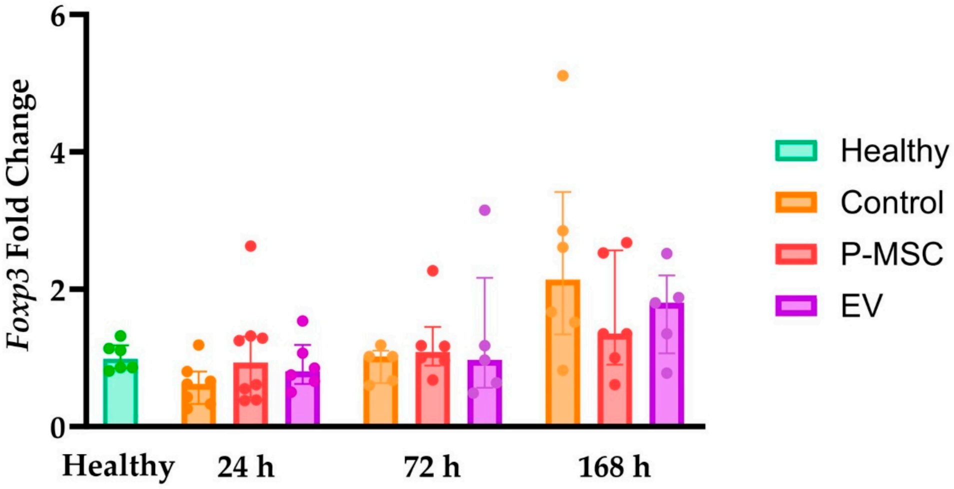

Supplementary Figure S3. *Foxp3* gene expression. Gene expression changes are presented as fold changes in comparison with tissue from healthy mice. P-MSC - human placental stem cell-treated, and EV - P-MSC-derived EV-treated. Individual data points are shown. P-MSC - human placental stem cell-treated, and EV - P-MSC-derived EV-treated. Columns represent median values with 25th and 75th quartiles as error bars.
